# Land Cover Classification from Very High-Resolution UAS Data for Flood Risk Mapping

**DOI:** 10.3390/s22155622

**Published:** 2022-07-27

**Authors:** Elena Belcore, Marco Piras, Alessandro Pezzoli

**Affiliations:** 1 DIATI, Politecnico di Torino, Corso Duca degli Abruzzi 24, 10129 Torino, Italy; marco.piras@polito.it; 2 DIST, Politecnico and Università degli Studi di Torino, Viale Mattioli 39, 10125 Torino, Italy; alessandro.pezzoli@polito.it

**Keywords:** UAS, land cover, very high resolution, structure from motion, machine learning, climate change, floods, SDGs, Sendai framework

## Abstract

Monitoring the world’s areas that are more vulnerable to natural hazards has become crucial worldwide. In order to reduce disaster risk, effective tools and relevant land cover (LC) data are needed. This work aimed to generate a high-resolution LC map of flood-prone rural villages in southwest Niger using multispectral drone imagery. The LC was focused on highly thematically detailed classes. Two photogrammetric flights of fixed-wing unmanned aerial systems (UAS) using RGB and NIR optical sensors were realized. The LC input dataset was generated using structure from motion (SfM) standard workflow, resulting in two orthomosaics and a digital surface model (DSM). The LC system is composed of nine classes, which are relevant for estimating flood-induced potential damages, such as houses and production areas. The LC was generated through object-oriented supervised classification using a random forest (RF) classifier. Textural and elevation features were computed to overcome the mapping difficulties due to the high spectral homogeneity of cover types. The training-test dataset was manually defined. The segmentation resulted in an F1_score of 0.70 and a median Jaccard index of 0.88. The RF model performed with an overall accuracy of 0.94, with the grasslands and the rocky clustered areas classes the least performant.

## 1. Introduction

Extreme floods are a severe natural threat for many sub-Saharan areas [1] and can cause conspicuous losses. In the last twenty years, many countries of the Niger basin have suffered from the effects of heavy rains and devastating floods [2,3,4]. In these areas, complete flood risk assessments are particularly important for risk management and prevention [5].

Indeed, the United Nations (UN) recognize the importance of planning against climate change-induced hazards to more effectively protect persons, communities, and countries, their livelihoods, health, cultural heritage, socioeconomic assets, and ecosystems [6]. Thus, monitoring the world’s areas that are more vulnerable to natural hazards has become crucial worldwide. In order to reduce disaster risk, effective tools and relevant data are needed. According to the agreement on Disaster Risk Reduction in the United Nations’ document, the Sendai framework [6], the Earth observation programs and in situ information should be promoted by encouraging tools for managing geographic data, such as the Geographic Information Systems (GIS).

The Sendai framework is a 15-year, voluntary, non-binding agreement which aims for the substantial reduction of disaster risk and losses in lives, livelihoods and health and in the economic, physical, social, cultural and environmental assets of persons, businesses, communities and countries [7]. Inadequate flood resilience can weaken or even reverse progress toward the Sustainable Development Goals (SDGs). Indeed, disaster risk reduction (DRR) and sustainable development are inextricably linked and interconnected [8]; all SDGs implicitly focus on reducing or monitoring risks caused by climatic variations. The Sendai framework’s targets one, two and three focus, respectively, on substantial reductions in (i) disaster mortality, (ii) the number of affected people, and (iii) direct economic losses. The SDGs and the Sendai framework are so interconnected that the UNDRR (United Nations Disaster Risk Reduction) developed shared indicators [8].

In this framework, it is fundamental to understand the deep connection between geophysical hazards and land cover (LC). The LC maps provide essential data to describe the flood risk and develop flood risk management plans [9,10]. On the one hand, the land cover influences the flood itself. Covers such as urban areas, bare soil, arable lands, and shrubs have differing permeability. Consequentially, the dominance of one over the other, or generally an unbalance in their distribution, strongly affects flood behavior. The land cover also defines the surface roughness, which plays a crucial role in the runoff. For example, Booth et al. [11], demonstrate that grass-dominated landscapes exhibit larger peak flows than forest-dominated landscapes with impervious areas. The increase in the runoff in a water basin is a direct consequence of reducing the discharge time and, therefore, of the possibility of floods [4].

On the other hand, mapping the LC provides information regarding the items exposed to flood risks, such as infrastructure, agricultural land, and human settlements; although, as Siejka et al. [5] underline, the land cover in the vicinity of rivers is rarely taken into account in assessing the flood risk. When considered, it is often detected from the photointerpretation of low spatial-resolution data or semi-automatic classification algorithms. Nevertheless, highly detailed information can add further information by detecting the items particularly endangered by floods; for example, the houses built with non-water-resistant material, as in southwest Niger, where traditional houses are made of earth and wooden poles.

One of the reasons behind the infrequent use of UAS information for LC is that automatic classification can be complex due to the high radiometric heterogeneity and the low spectral resolution. Object-oriented classification methods are generally more efficient in classifying the land cover for images with very high resolution, such as those captured using unmanned aerial systems (UAS) systems. Although UAS data usually have low spectral resolution and, inevitably, the features to be classified have a relatively similar spectral response, they can be separated using spatial, texture, and contextual information [9]. A typical case is represented by concrete roofs and parking lots, which usually have a similar spectral signature but differ in texture and shape. The limitations of low spectral detail are often reduced by incorporating texture features, elevation features, and robust classifiers.

Several studies used different approaches and models for land cover classification from VHR optical data. These studies have primarily varied according to the technique, training sample size, and input dataset. Zhang et al. [12] developed a classification framework based on deep learning and object-based image analysis (OBIA) that classifies UAS RGB imagery with 5 cm resolution into five categories. They use spatial, spectral, and textural features for 280 training objects. The classification achieved an overall accuracy of 97%. Furthermore, Trevisol et al. [13] developed an OBIA classification of roof coverings based on texture, achieving very high accuracy with 10 cm resolution in urban areas.

Similarly, Sameen et al. [14] classified an RGB orthomosaic with a 10 cm resolution of urban areas using a convolutional neural network (CNN). They achieved promising results, although the training dataset was pretty large compared to one of the other research papers, and they identified seven classes with relatively low thematic detail. Liu and Abd-Elrahman [15] propose seven high-thematic detail category classifications of wetlands from six-centimeter resolution UAS imagery using multi-view information. They used 2800 training objects and reached an overall accuracy of 80.4%. A novel method was developed by Kalantar et al. [9] that uses a fuzzy unordered rule. Their method achieved a 91% overall accuracy. Other authors investigated the optimization of UAS imagery classification, such as Ma et al. [16], who researched the optimum number of input features in the random forest (RF) and support vector machine (SVM) classification of UAS RGB imagery (with 0.2 m resolution). They identified with the RF classifier eight features for 300 training objects. De Luca et al. [17] identified five classes from RGB and NIR information, achieving 97% overall accuracy using the RF algorithm and 150 objects for each class. Most of these studies analyzed RGB imagery. Even though they generally obtain good results, they considered few classes with relatively low thematic detail. Moreover, none of them analyzed the quality of the segmentation from a geometric point of view.

This work aimed to identify nine high-thematic classes from an RGB-NIR dataset using supervised OBIA-RF classification. Particular attention was given to the definition of classes to satisfy the flood risk management requirements and correctly identify the flood-exposed buildings from a geometrical point of view. A geometric validation for the segmentation was carried out for the buildings. Moreover, this research tried to overcome the critical aspects of LC mapping in the Sirba basin using low-cost geomatics. Potentially flooded areas were mapped using images collected via UASs with low-cost optical sensors.

## 2. Materials and Methods

### 2.1. Study Area and Contextualization

**Figure 1:**
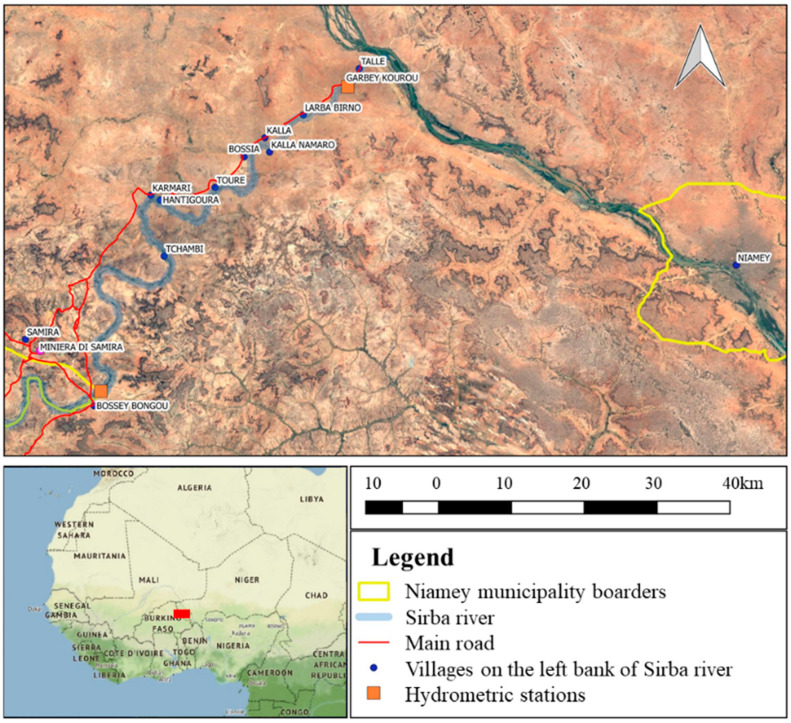
Sirba River in Niger.

Over three-fourths of the Sahara has an annual average rainfall of less than 100 mm/m^2^. One-fourth has less than 20 mm/m^2^. In these areas, local enhancements of temperature and heavy rains occasionally occur [18] owing to the sudden change of weather patterns that bring more extreme weather events, of which the flash floods of the dry valleys are the most devastating [19]. Floods in the Sahara are often characterized by deep, fast-flowing water, which, combined with the short time available to respond, increases the risk to people and property [20]. In the last decades, there has been recorded evidence of an increasing number of massive rainfall events over the West Sahel [21,22]. The climatic conditions of the Sahelian zone of the Niger basin are not an exception. They fit the changing climate pattern of the Sahel. Catastrophic flooding has become an increasing threat during the last decades, affecting more than ten million people since 2000 [23]. The Sirba River, a tributary of the Niger River, crosses Burkina Faso and Niger (Figure 1). The Sirba basin is prone to floods, and villages along the river are vulnerable to economic and human losses [4,24,25].

Along the Nigerian branch of the Sirba (about 100 km length), the only existing route connecting the upstream villages to the capital (Niamey) is often flooded, complicating any movement during the rainy season. The Sirba basin is 39,138 km^2^, and direct monitoring is almost impossible due to the vastness of the area, the difficult climatic conditions, and the lack of a developed road network. In this area, there is an urgent need for climatic planning and the development of adaptation strategies to climate change at the local level [26]. Despite this undeniable need, there is no appropriate risk mapping of the area; indeed, subnational risk mapping lacks detail [26]. The Sirba basin in Niger territory is the study area of the ANADIA 2 project. The Italian Agency for Development and Cooperation (AICS) funds it [24]. The project aims to create an early warning system to face climate change effects in Sirba River Basin (The transboundary basin of Sirba river lies between the countries of Burkina Faso and Niger in the centre of the Sahel strip. It is a sparsely populated area whose inhabitants are dedicated to food and farming activities.), enhance local technicians’ knowledge regarding flood forecasting, and create an adaptation strategy plan for a village along the Sirba River.

**Figure 2:**
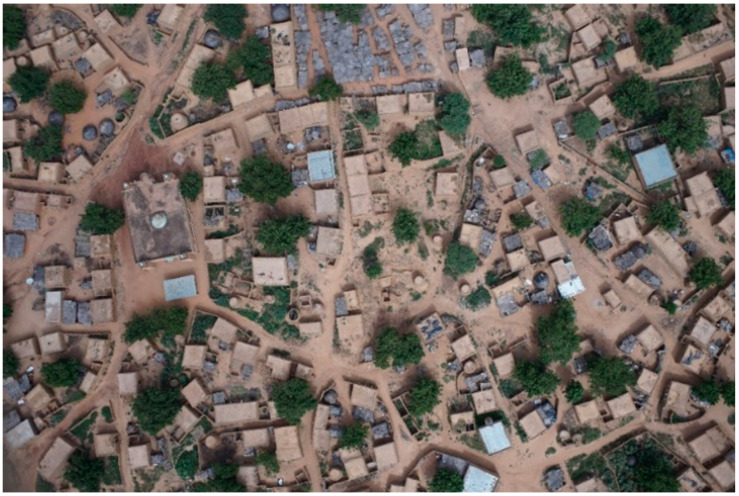
Example of local architecture. Nadiral view.

The data gathered and the information provided by this research are directly involved in ANADIA 2.0, by feeding the adaptation strategy plan and investigating the cause–effect relation of floods. The village of Touré is interested in adaptation-planning strategy, it is the object of very high-resolution unmanned aerial systems (VHR-UAS) analysis. The difficulty of land cover mapping in this area is due to the high spectral homogeneity of the cover types. One of the most significant problems in the remote sensing of sub-Saharan regions is that reflectance from soil and rock during the dry season is often much greater than that of the sparse vegetation, making it difficult to be separated from the other classes. Some of the specific problems involved with remote sensing of arid vegetation include the multiple scattering of light (nonlinear mixing) between vegetation and soil [27]. Moreover, it is hard to separate built-up areas from soil even from VHR imagery. Most of the buildings along the Sirba River are made of locally produced brick. This production is realized using clays from the Sirba riverbed and the buildings are not plastered (Figure 2).

The main road and streets of the village are unpaved. As a consequence, the spectral response of buildings is the same as that of roads and bare soil. The strong seasonality adds further complexity to the classification as the frequent cloud cover during the rainy season and sand presence in the air may alter the sensed data’s spectral values. From the geodetic point of view, the Sirba River area is disadvantaged. A dense network of permanent stations is not available to process GNSS data [28,29]. Despite CORSs covering most of the world’s countries today, some areas are still not included in the network, such as some sub-Saharan countries and the Sirba basin. Indeed, the lack of CORS and known coordinates points is quite a frequent condition in sub-Saharan rural areas, strongly affecting topographic surveys. Indeed, there is poor access to general services (e.g., electricity, computers) and few people with enough expertise to use GNSS software. These unfavorable conditions can be exacerbated by emergencies, such as during (or immediately after) natural hazards.

### 2.2. Data Collection

#### 2.2.1. UAS Flights

**Figure 3:**
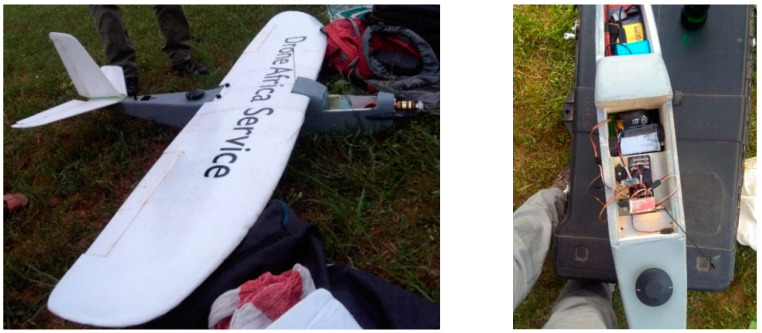
**Left**: UAS system built by Drone Africa Service. **Right**: Detail of the UAS body. It is visible that the Raspberry Pi sensor is mounted on the UAS system; 3.5-inch screen and the GPS receiver.

Two optical sensors mounted on a fixed-wing unmanned aerial system (UAS) collected the data. Specifically, one sensor was sensitive to the visible (red-green-blue, RGB) part of the electromagnetic spectrum, and one sensor to the infrared (near infrared-green-blue, NGB). Using a GNSS dual-frequency receiver, the ground control points (GCPs) for the precise georeferencing of the UAS imagery were collected. The UAS system was provided by a Nigerien enterprise based in Niamey, Drone Africa Service (DAS). DAS uses self-constructed and no-brand drones. A fixed-wing UAS was built explicitly by DAS to be used in this survey (Figure 3). The flight was planned and automatically controlled by the ArduPilot software. Two optical sensors were mounted on the system: a Sony ILCE-5100 camera and an experimental sensor created with a Raspberry Pi computer and two Raspberry Pi 2 cameras, as described in Belcore et al. [25]. The Sony ILCE-5100 is a mass-market mirrorless RGB digital camera. It has an APS-C type Exmor CMOS sensor to collect three-band imagery. It has a 23.3 MP resolution and 1.5× multifocal length. The sensors were not simultaneously used because they were too heavy to be held up together by the UAS during the same flight. Furthermore, they have different characteristics, and each camera requires specific flight settings (i.e., height and speed of flight) to ensure imagery of similar ground sample distance (GSD) and the same overlap between the pictures.

**Figure 4:**
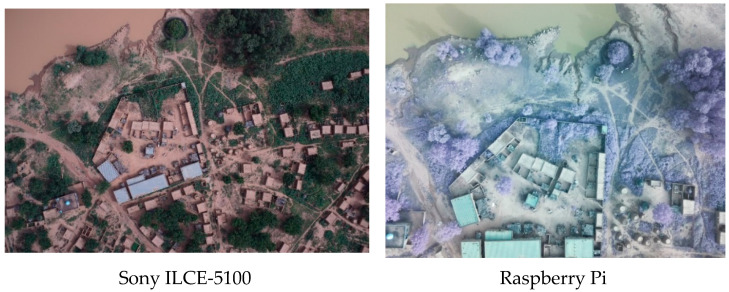
Sample pictures of the same area acquired by Sony-ILCE-5100 and Raspberry Pi. The SONY picture (6000 × 3000 pixels) has a 3.9 cm/pixel resolution and is in RGB. The Raspberry picture (2592 × 1933 pixels) has a 6.1 cm/pixel resolution and is visualized in red-green-NIR false color composition.

Table 1 presents the characteristics of the flights. The Sony camera collected 507 images with 3.91 cm/pixel GSD in about 30 min of flight at 280 m above the ground. The Raspberry camera allows the collection of near-infrared (NIR) information and acquires information with 5-megapixel resolution. To obtain a GSD of 6.1 cm/pixel images, it was necessary to reduce the height of flight to 130 m above the ground. To cover the same area of the Sony flight, two flights of about 30 min were necessary, which is the maximum duration of the UAS battery. Three UAS flights covering a surface of about 300 hectares were performed in one day [30]. In particular, 280 hectares were covered; the study area included urban areas and rural areas surrounding the village. Some 636 pictures were collected with the Raspberry NIR, and 507 with the Sony (Figure 4).

A measuring campaign using two GNSS dual-frequency receivers, STONEX S10 models, in RTK rover-base modality was performed for georeferencing the data. In an RTK rover-base survey where the station master coordinates are unknown, two receivers are used: a GNSS receiver works as master station, storing the satellite-based measure of its position with a specific frequency (in this case, the rate was set to 1 Hz) and a rover receiver, connected to the master receiver (during the Sirba survey the transmission was via radio) measures the points of interest based on its position concerning the master station. The master station coordinates were then estimated in post-processing using the precise point positioning (PPP) technique. The Z component was recorded in ellipsoidal heights and converted into orthometric heights using the EGM08 model [31]. In Tourè, 16 points were measured.

#### 2.2.2. Orthophoto and DEM Generation

The data collected were processed according to a traditional structure from motion (SfM) workflow [32]. The results were one digital surface model (DSM) and two multiband orthomosaics in red-green-blue and red-green-near infrared. To generate the digital models for the LC classification, all images were imported in Agisoft Metashape Professional (AMP), a commercial structure for motion (SfM) software for photogrammetric block processing [33]. The images were subdivided into two different chunks, one for each sensor. The images in each frame were then aligned using the generic preselection option and based on image pixel characteristic correspondences between two or more superimposed frames, it resulted in a sparse point cloud [34]. To generate the clouds, the “High” level of accuracy was set for all the processed chunks and tie points limited to 40,000. Since the sparce point clouds were obtained without a reference datum, the points measured with GNSS receiver were used as ground control points (GCPs) to georeference the entire photogrammetric block. Specifically, 10 GNSS points were used as GCPs and 6 as control points (CPs) for the validation of the precision achieved in the georeferencing phase. The GCPs were measured in all frames in which they had been found and used to optimize the camera’s interior parameter estimation and to improve the generation of the photogrammetric block. Then, the chunks were aligned based on the GCPs location. Table 2 shows the final RMSE (root mean square error) values obtained for GCPs and CPs.

Subsequently, “high quality” dense point clouds were generated for each chunk. The dense point cloud is the source product for the generation of the digital elevation model and the orthomosaic.

Then an interpolated mesh of the point cloud was created, the texture, and finally, the orthomosaic. The same process was followed for the chunks acquired by the Raspberry Pi camera and the Sony ILCE. The final orthophotos had a resolution of 4 cm in RGB and 6 cm in NGB and were aligned according to the markers (approximately 3 cm precision). The Sony ILCE-point cloud was used to extrapolate the DSM because of its higher spatial resolution than the Raspberry-point cloud. For each chunk, the dense cloud was cropped into a “high interest” area that coincided with the village’s centre. The cropped dense cloud was classified using the ground classification tool inbuilt in Metashape to identify the ground points. The classification was automatic and based on three parameters: maximum degree angle, maximum distance, and cell size. Table 3 shows the settings used for the ground classification.

The ground points were used for generating the DTM with 7.81 cm/pixel resolution and 164 points/m^2^; also, a digital surface model (DSM) was computed with the same resolution. The normalized digital terrain model (*nDTM*) of pixel i, j was computed as the difference between DSM and DTM, by using Equation (1).

(1) $$nDTM_{i,j}={\mathrm{DSM}}_{i,j}-{\mathrm{DTM}}_{i,j}$$

#### 2.2.3. Classification System

**Figure 5:**
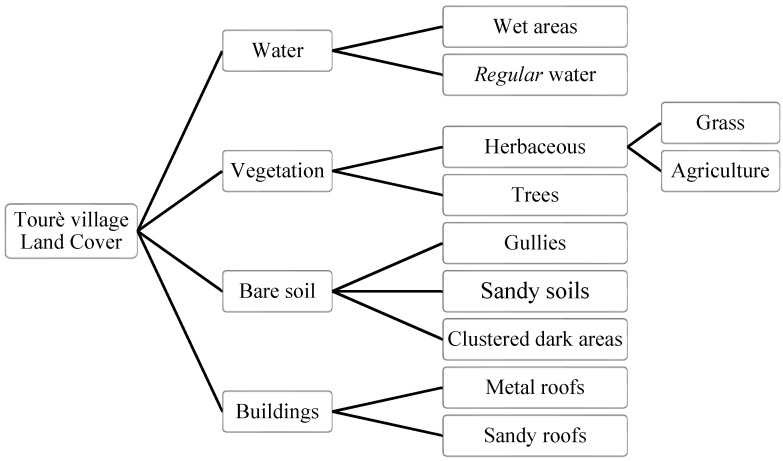
Classification legend structure. It is composed of 4 macro-classes (buildings, bare soil, vegetation, and water); nine classes and two sub-classes (grass and agricultural land).

The classification system consisted of four macro-classes (water, vegetation, bare soil, and buildings), nine classes, and two sub-classes (Figure 5).

The macro-class “buildings” is divided into two classes according to the material of the roofs: brick roofs (or sandy roofs) and metal roofs. The separation of the roof types is due to two reasons. First, these classes are differently affected by floods; indeed, houses with metal roofs are generally built with concrete bricks (as per the authors’ experience) and thus less prone to be damaged by floods. In contrast, sandy brick roofs are typical of traditional building techniques that consist of sun-dried bricks sustained by wooden poles and kept together by mud. Floods significantly damage these buildings. Even though they are very similar from a categorical point of view, the two classes strongly differ in the spectral response. Similar reasons exist behind the choice of the bare soil classes. Indeed, each type of soil has specific permeability and roughness that floods impact differently. Each soil type within the study area has a specific spectral response that differs from other soils’ spectral responses. This is particularly true for the red soils of the gully class and the sandy ones in the study area. The class of agricultural land and the buildings are a significant class for the risk management plan. Agriculture is the main source of income for the local population, and the damage of agricultural fields would be a consistent economic loss that can affect the food security of many households. Finally, the wet lands class is another class of interest for flood risk management because it represents an extremely variable class; it allows the analyst to define the limit of watered areas.

#### 2.2.4. Features Extraction and Segmentation

The input dataset was enriched with derivative features, namely, spectral, textural, and elevation-based features. Ten features were extracted from the RGB and RGN datasets and used for the segmentation process: two spectral features, one edge-extractor feature, five grey level co-occurrence matrix (GLCM) textural features, and one digital surface model. GLCM is calculated on subgroups of pixels or an n × n filter. In GLCM, the number of columns and rows is estimated based on the number of grey levels in the image [35,36]. The GLCM matrix elements show the statistical estimated values that happen between the grey-level value of a pixel at a special direction θ and distance d [36]. Based on the grey level co-occurrence matrix, Haralik, in 1973 [36], proposed 14 measures (angular second moment, contrast, correlation, sum of squares, inverse difference moment, sum average sum variance, sum entropy, entropy, difference variance, difference entropy, information measure of correlation, maximal correlation coefficient), which have been broadly applied on land cover classification studies and are now implemented in most of the existing classification software. The semantic segmentation was carried out in three steps, each of which focused on identifying specific objects (“Buildings” and “Trees”). First, the dataset was segmented with a multiresolution algorithm based on the GLCM data a and the NIR information, then the class buildings was assigned to the object that satisfied four conditions: nDSM value larger than 4 m; mean difference of mean nDSM to the neighbors larger than 0.2 m (to avoid confusion with trees) and NDWI and GLCM specific thresholds identified by iterative tests. The remaining objects were then re-segmented and separated in two classes according the NDWI values in vegetation and non-vegetation. Subsequently, vegetated areas were segmented according the nDSM and the height difference to neighbors objects to correctly segment trees and herbaceous vegetation. Table A2 of Appendix B shows the eCognition ruleset used for the segmentation. The parametrization of the ruleset is based on an iterative process and the authors’ experience. The classes assigned during the segmentation procedure are considered “service classes” and not used during the RF classification. Service classes are assigned according to simple and often imprecise rules, for this reason they are not included in the classification system.

The OBIA approach allows the analyst to introduce into the classification model features regarding the segmented objects’ geometric characteristics and their relationship with the neighborhood’s objects: 39 features were computed for each object. Table A1 in Appendix A reports the used measures for segmentation and classification.

#### 2.2.5. Training Selection and Classification Model

The training and test dataset comprised 1786 objects: 200 sample objects for each class, except for classes 9 (gullies) and 10 (metal roofs) which have, respectively, 108 and 78.

The difference in the number of objects reflects the classes covered within the study scene and the objects’ size. The training and test objects were randomly selected within the scene using Qgis *Random points in extent* algorithm. The objects on which the points lay were manually labelled, and, finally, to balance the number of samples between the classes, some samples were manually added. The samples were used to train the model in proportion 50–50, as Table 4 shows.

The model was trained in an eCognition environment, with 100 depth, 4 minimum sample count, and maximum tree number 50. The parametrization was selected according to the authors’ experience and the literature on RF in OBIA and LC classification [37,38,39].

#### 2.2.6. Accuracy Assessment

**Figure 6:**
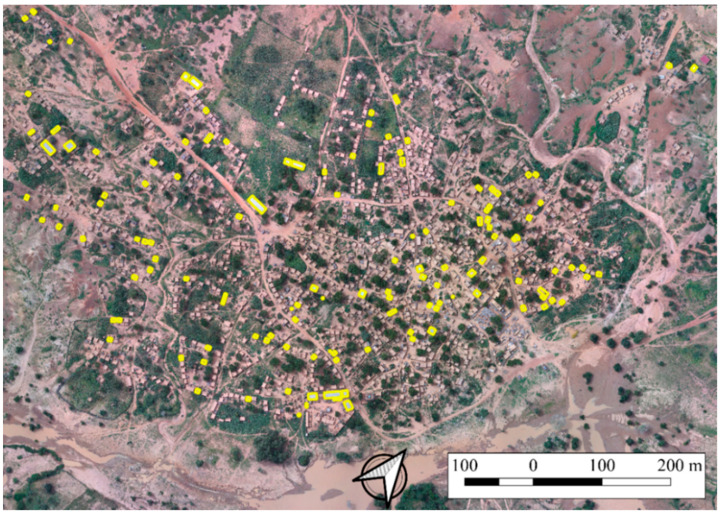
Position of the reference objects within the study area.

The accuracy of the segmentation was evaluated with specific metrics only for the class “Buildings”, since its geometric definition and position have been considered by the planners more important in flood risk management than the other classes. Namely, visual evaluation and quantitative metrics were realized, in indices and RMSE both forms. The validation used for the segmentation was a two level-accuracy assessment. The first level is based on [40], and it consists of a simple visual evaluation. The second level assessment is a quantitative method that compares several variables, and it assesses the under-segmentation and over-segmentation. Both accuracy assessment levels used as reference 133 objects randomly selected, but manually delineated (Figure 6).

The accuracy was evaluated in terms of correspondence between the reference houses and the segmented ones. The quality of the segmentation was assessed in two steps.

**Figure 7:**
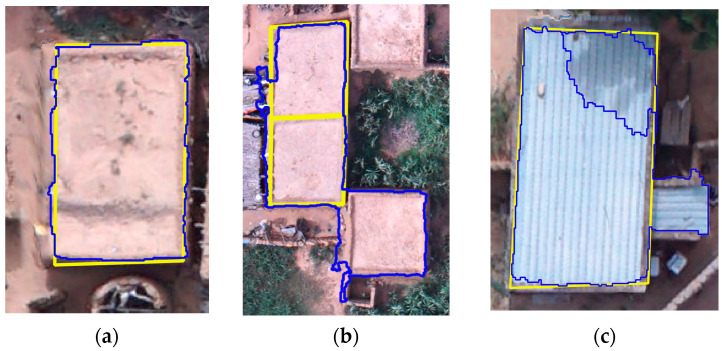
Possible relations between reference objects (yellow outline) and segmented objects (blue outline). (**a**) Match. (**b**) Omission through under-segmentation. (**c**) Commission through over-segmentation.

Figure 7a shows matching objects (M), while the relations of reference and segmented objects in Figure 7b,c were considered as non-matching houses. The segmented houses were counted based on their overlap with the reference houses. For example, the segmented house in Figure 7c is one. Even if significant, these measures provide a partial view of the quality of the segmentation. The omission and commission errors can describe more precisely the quality of the segmentation. As illustrated by [40], three possible cases of the relation between the reference dataset and the segmented one were taken into consideration: (i) match, (ii) omission through under-segmentation, and (iii) commission through over-segmentation (Figure 7).

As quantitative for quality of the segmentation, the areal difference, the perimeter, the centroid’s distance, the over-segmentation index (*OS*) (Equation (2)), the under-segmentation index (*US*) (Equation (3)), the completeness index (*D*) (Equation (4)) and the Jaccard index (J) (Equation (5)) were evaluated. The root mean square error (RMSE) was calculated for the area and the perimeter. The *OS* and *US* were adapted from Clinton et al. (2010) and Persello (2010) [41,42].

(2) $$OS=1-\frac{\left|R_{i}\cap S_{i}\right|}{\left|R_{i}\right|},$$

(3) $$US=1-\frac{\left|R_{i}\cap S_{i}\right|}{\left|S_{i}\right|},$$

(4) $$D=\sqrt{\frac{OS_{i}{}^{2}+US_{i}{}^{2}}{2}},$$

(5) $$J=\frac{\left|R_{i}\cap S_{i}\right|}{\left|R_{i}\cap S_{i}\right|}$$.

Their estimations are based on the relation between the segmented (S) area and reference objects (R), where $R_{i}\cap S_{i}$ in the overlapping area between the reference crown ($R_{i}$) and the segmented crown ($S_{i}$) of object *i*.

The classification quality was evaluated using the overall accuracy, producer’s accuracy, user’s accuracy, and F1 score metrics.

## 3. Results

**Figure 8:**
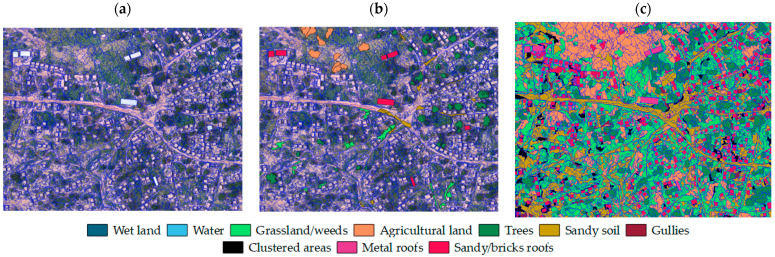
(**a**) Example of segmentation in a sample area, (**b**) training samples and (**c**) classification.

The complete segmentation was realized in 11 h (These data should be considered carefully, since the analysis was machine-dependent. It was realised using a laptop with 16 GB RAM and 2.7 GHz processor.), and was composed of 34,439 objects, while the classification was carried out in approximately 4 h, Figure 8.

The visual assessment revealed a tendency to over-segmenting (14 over-segmented objects against 7 under-segmented objects), Table 5. Of the houses, 84% were detected (112 matches over 133 references). The F1 score, which related user’s and producer’s accuracies, was 70%.

The area-based measures did not confirm the tendency of the segmentation method to over-segmenting. The median value on the over-segmentation average was only 0.32, while the under-segmentation index was 0.63. Although the difference between the indices is small, it is unneglectable. The completeness index (D) provided extremely positive information (0.069) along with the Jaccard index, which was 0.88 and reflected the F1 score of visual assessment (Table 6).

Table 7 reports the RMSE measures, which indicate the precision of the extension of the segmented houses over the reference ones. The error over the area was, on average, 2.29 m^2^, which represents the 6% on the average house extension. In contrast, the RMSE of the perimeter was slightly higher, and reached 18% of error over the average perimeter extension.

**Figure 9:**
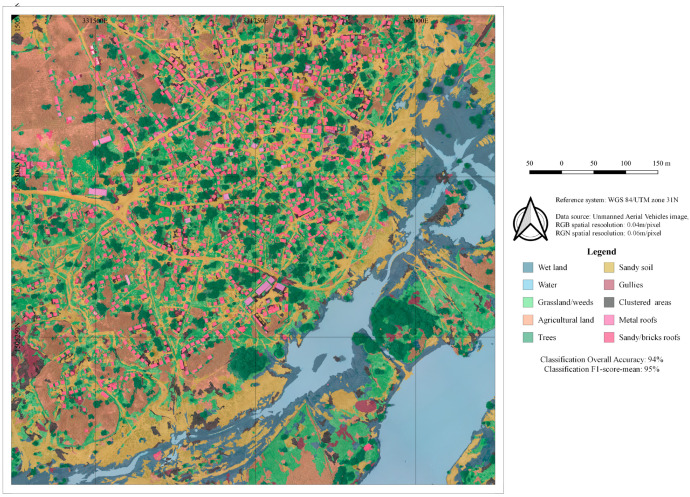
LC map of Tourè.

The overall accuracy was over 94% (Table 8). Except for grassland and dark clustered rocky soils, classes that have relatively low accuracy, the classes have a user’s accuracy of over 90%. Figure 9 shows the final LC map.

## 4. Discussion

The assessment of the houses’ segmentation provided outstanding results. Indeed over 84% of the houses were correctly detected, and a low number of objects were under-segmented. Over-segmentation was a frequent error, although apparently, the most common condition was the correct segmentation of the houses in terms of extension (as testified by the low RMSE aver the area, 6%), described by more than one object. This may be caused by the roofs’ spectral variation due mainly to shadows or damaged roofs, or objects on the roofs. Figure 7c is an example of over-segmentation caused by shaded roofs. Furthermore, it appeared from visual interpretation of the aerial images that some households stock wood/straw covered by black plastic sheets on the roofs, interfering with the segmentation process. This frequent condition may also explain the higher RMSE over the perimeter, in respect of the area. Even if the over-segmentation demonstrates the segmentation to be less accurate, it is worth mentioning that it was partially overcome in the classification phase since the different segments were classified as “buildings” and later merged.

**Figure 10:**
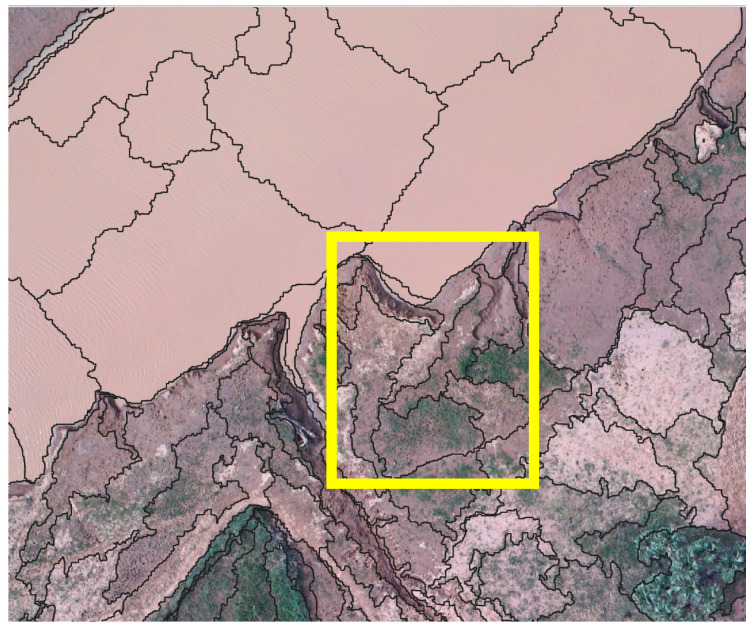
Detail of the segmentation process. The yellow square indicates two objects of mixed class: grassland and wet areas.

In regard to the classification, the random forest algorithm provided accurate results. The class grassland was seldom classified as agricultural land and wet areas. The confusion between grassland and agricultural land can be explained by the spectral similarity between them and the lack of textural distinction. It is worth underlining that some agricultural land does not have the typical cultivation pattern of plants in lines, thus it is not always detected by the textural information. At the same time, the confusion between grassland and wet areas depends on the presence of transition cover between the two, as Figure 10 shows.

**Figure 11:**
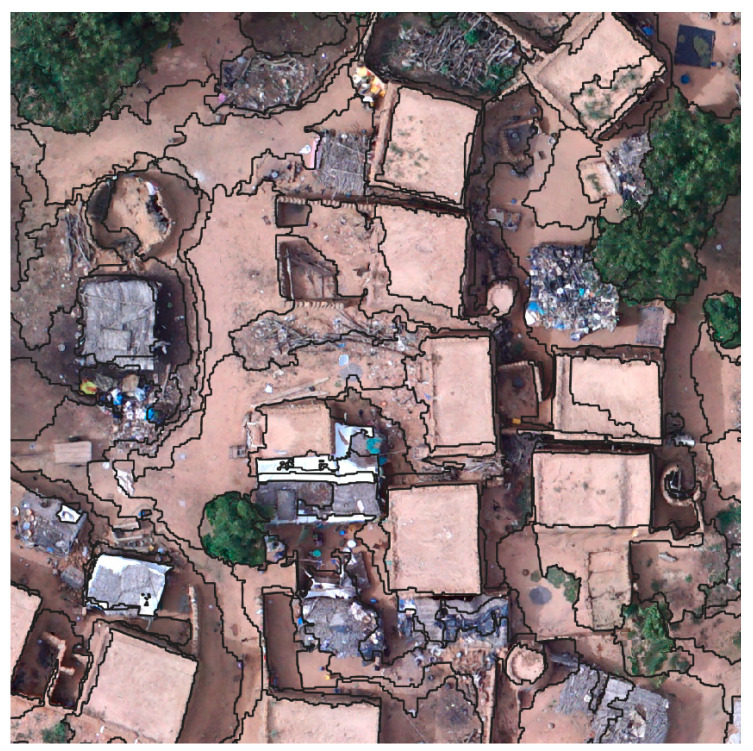
Example of land cover spectral variability within households’ yards.

The clustered rocky soil was confused with gullies. The main feature that distinguishes them is the nDSM and its difference from the neighboring objects. As with the wet areas and the grassland, some mixed classes between sandy and clustered soils exist. The main challenges faced during the classification were related to two main aspects: the high variability of the land cover within the village and the class definitions. The first is related to various objects in household yards that are part of everyday life, such as in Figure 11 (pot, dishes, plastic sheets, bottles, etc.). These objects have a significantly different spectral response since they are made of various materials, and thus, they create noise in the classification, altering the spectral values and the texture of these areas. Basically, this class is not fully represented in the classification system, and it was classified as bare soil. The other challenging aspect was regarding the confusion between agricultural areas and grasslands. The two classes are separated mainly by textural features based on the regular distribution of plants in rows. Nevertheless, this is not always true, some plots are rich in weeds, and the regular row pattern is imprecise since the agricultural work is not mechanized.

It is worth underlining that, according to the literature, the training dataset was relatively small, only 100 samples described each class, but it was revealed to be sufficient for acceptable results. Similarly, some classes were underrepresented (fewer training objects), although this imbalance mirrors the area’s real cover conditions. Such imbalances in the training datasets must be considered normal for the classification of relatively small areas and very specific classes such as the gullies and rocky-clustered areas. The same imbalance was present on the test dataset. Apart from the inaccuracies related to the particularity of the area, the classification provided good results for DRR plans.

## 5. Conclusions

This work aimed to define high-thematic resolution LC classes related to flood hazard risk reduction planning. The LC was mapped using OBIA classifications based on random forest classifiers. Data were collected via UASs using low-cost and open NIR optical sensors.

The increase in the spatial and thematic resolution was directly proportional to the need for additional input features and the textural information was revealed to be fundamental in the classification and segmentation phases. Indeed, the GLCM-derived measures were revealed to have a strong influence on the segmentation and classification processes. Due to the high-spectral heterogeneity of VHR imagery, the single pixel of a specific feature of the scene can have enormous spectral variation. This is the main reason behind the spread of OBIA techniques in their classification. The segmentation is still strongly dependent on the analyst’s experience, and many different approaches exist. Although the final accuracy of the classification is acceptable for the risk reduction plan, additional research needs to be realized to find shared and efficient segmentation approaches.

The final classifications satisfied the planners’ needs and partially overcame the criticalities related to the high spatial resolution and high cover heterogeneity of the study area. The final accuracy is acceptable for disaster risk planning and it allows the precise quantification of the exposed infrastructures and estimation of the potential flood-induced losses in the area.

## Figures and Tables

**Table 1:** Main characteristics of the sensors and the flights performed at Tourè.

Characteristics	Sony ILCE-5100	Raspberry PI
Resolution	23.3 MP	5 MP
Band sensor	RGB	RGBN
ISO settings	1/125	1/100
Shutter frequency	Automatically set by the navigation software	1 Hz
Lateral overlap	70%	70%
Longitudinal overlap	60%	60%
Number of flights	1	2
Average duration of flights	30 min	30 min
Height of flight from the ground	280 m	130 m
GSD	3.9 cm/pixel	6.1 cm/pixel

**Table 2:** RMSE value of the ground control points (GCPs) and check points (CPs).

Errors (cm)	GCPs		CPs	
	Sony ILCE RGB	Raspberry RGN	Sony ILCE RGB	Raspberry RGN
X error-easting	3.52	5.40	3.75	5.41
Y error-northing	3.77	5.05	3.81	6.54
Z error-altitude	3.79	2.93	7.90	3.03
Total error	6.40	7.95	5.67	9.02

**Table 3:** The parameter set of the ground classification algorithm. Maximum degree angle describes the maximum slope of the study area expressed in degrees; maximum distance is the maximum distance between the ground and the highest feature in the scene; cell size is the size of the most extended cell in which there are no detectable ground points (i.e., very dense woods, big buildings).

Parameter	Value
Maximum degree angle [degree]	1.5
Maximum distance [meters]	25
Cell size [meters]	30

**Table 4:** Number of sample objects of training and test datasets.

No. Samples	Wetland	Water	Grassland	Agricultural	Trees	Sandy Soil	Clustered Dark Areas	Gullies	Metal Roofed Houses	Brick Roofed Houses
Training	100	100	100	100	100	100	100	54	39	100
Test	100	100	100	100	100	100	100	54	39	100

**Table 5:** Visual assessment metrics of the segmentation of the buildings in the Niger case study.

Visual Validation	No. Objects
No. References	133
No. Segmented	185
Matches	112
Omission through under-segmentation	7
Commission through over-segmentation	14
Producer’s accuracy	0.842
User’s accuracy	0.605
F1 Score	0.704

**Table 6:** Area-based quantitative assessment of the segmentation of the buildings in the Niger case study. * Lower values means better segmentation.

	Over Segmentation Index *	Under Segmentation Index *	D *	Jaccard Index
Average	0.063	0.122	0.113	0.830
Min	0.000	0.002	0.009	0.181
Max	0.473	0.786	0.560	1.000
Median	0.032	0.063	0.069	0.882

**Table 7:** Root mean square error of the area and perimeters of the house objects in the Niger study area.

Metric	RMSE	Average Value	Percentage over the Total
Area [m^2^]	2.289	40.594	6%
Perimeter [m]	4.368	24.778	18%

**Table 8:** Error matrix of object-bases classification of Tourè along the Sirba River (Niger).

	Wetland	Water	Grassland	Agricultural	Trees	Sandy Soil	Clustered Dark Areas	Gullies	Metal Roofs Houses	Bricks Roofs Houses	OA
PA	0.926	1.000	0.966	0.933	0.971	0.912	0.956	0.902	0.978	0.923	0.945
UA	1.000	0.980	0.850	0.970	0.980	0.930	0.869	0.937	1.000	0.960	
F1	0.962	0.990	0.904	0.951	0.975	0.921	0.910	0.919	0.989	0.941	

## Data Availability

The data presented in this study are available on request from the corresponding author.

## Appendix A

**Table A1:** Features selected for the segmentation and the classification.

Feature Group	Feature Name	Note	Software	Segmentation	Classification
Spectral	Normalized Difference Water Index (NDWI)	(McFeeters, 1996)	Orfeo toolbox	X	X Mean value
	Enhanced Vegetation Index (EVI)		Orfeo toolbox	X	X Mean value
	HUE	Calculated on RGB	eCognition		X
	HUE	Calculate on NIR	eCognition		X
	Normalized Difference Water Index (NDWI)		eCognition		X Standard deviation to neighborhood
	Enhanced Vegetation Index (EVI)		eCognition		X Standard deviation to neighborhood
	Brightness		eCognition		X
Edge-extractor	Sobel		eCognition		X Mean value
	Sobel		eCognition		X Standard deviation to neighborhood
Textural [36]	Grey Level Co-occurrence Matrix (GLCM) Sum Variance	Calculated on NIR channel	Orfeo toolbox	X	X Mean value
	Grey Level Co-occurrence Matrix (GLCM) Dissimilarity	Calculated on Green Channel	Orfeo toolbox	X	X Mean value
	Grey Level Co-occurrence Matrix (GLCM) Sum Average	Calculated on Green Channel	Orfeo toolbox	X	X Mean value
	Grey Level Co-occurrence Matrix (GLCM) Sum Variance	Calculated on Green Channel	Orfeo toolbox	X	X Mean value
	Grey Level Co-occurrence Matrix (GLCM) Dissimilarity	Calculated on NIR channel	Orfeo toolbox	X	X Mean value
	Grey Level Co-occurrence Matrix (GLCM) Sum Variance		eCognition		X Standard deviation to neighborhood
	Grey Level Co-occurrence Matrix (GLCM) Dissimilarity		eCognition		X Standard deviation to neighborhood
	Grey Level Co-occurrence Matrix (GLCM) Sum Average		eCognition		X Standard deviation to neighborhood
	Grey Level Co-occurrence Matrix (GLCM) Sum Variance		eCognition		X Standard deviation to neighborhood
	Grey Level Co-occurrence Matrix (GLCM) Dissimilarity		eCognition		X Standard deviation to neighborhood
Elevation	Digital Surface Model	Calculated on RGB	/	X	X Mean value
	Digital Surface Model	Calculated on RGB	eCognition		X Standard deviation to neighborhood
	Slope				
RGB dataset	Red	/	/	X	X Mean value
	Green	/	/	X	X Mean value
	Blue	/	/	X	X Mean value
	Red	/	eCognition		X Standard deviation to neighborhood
	Green	/	eCognition		X Standard deviation to neighborhood
	Blue	/	eCognition		X Standard deviation to neighborhood
NIR dataset	Red_2	/	/	X	X Mean value
	Green_2	/	/	X	X Mean value
	NIR	/	/	X	X Mean value
	Red_2	/	eCognition		X Standard deviation to neighborhood
	Green_2	/	eCognition		X Standard deviation to neighborhood
	NIR	/	eCognition		X Standard deviation to neighborhood
Relation to neighbors	Mean difference to neighbors	Calculated on DSM	eCognition		X
Geometric	Length/width		eCognition		X
	Rectangular fit		eCognition		X
	Radius of the smaller enclosing ellipse		eCognition		X
	Compactness		eCognition		X

## Appendix B

**Table A2:** Segmentation ruleset applied for the Land Cover OBIA classification of Tourè village.

Algorithm	Parameters	Values	Computing Time	Layers (Weight) and Conditions
Houses				
Multiresolution segmentation	Scale parameter	60	1:19	DSM (1) GLCM_NIR_3 (1) Glcm_rgb_3 (2) Glcm_rgb_5 (1) Green_rgb (1) Nir (1)
	Shape	0.2		
	Compactness	0.8		
Assign class	Use class	Unclassified	0:27	Mean GLCM_adv_3_rgb >= 3.5 and Mean NDWI < 0.05 and Mean nDSM >= 4 And Mean diff. to neighbors DSM (0) >= 0.2 Mean
	Assign class	Houses		
Assign class	Use class	Unclassified	0:0.06	Rel. border to houses > 0.6
	Assign class	Houses		
Merge Region	Use class	Houses	0:0.04	
Multiresolution segmentation	Scale parameter	100	1:40	Only houses GLCM_NIR_3 (1) Glcm_rgb_3 (2) Glcm_rgb_5 (1)
	Shape	0.8		
	Compactness	0.2		
Trees				
Merge Region	Use class	Unclassified	0:03	
Multiresolution segmentation	Scale parameter	80	2:17	Only unclassified DSM (1) GLCM_NIR_3 (1) Glcm_rgb_3 (1) Glcm_rgb_5 (1) Green_rgb (1) NDWI (1) Nir (1)
	Shape	0.1		
	Compactness	0.5		
Assign class	Use class	Unclassified	0:21	Mean diff. to neighbors DSM (0) > 1 and Mean NDWI < 0.03
	Assign class	Trees		
Merge Region	Use class	Trees	0:01	
Grass				
Merge Region	Use class	Unclassified	2:55	
Multiresolution segmentation	Scale parameter	200	2:37	Only unclassified nDSM (1) Glcm_rgb_5 (1) Green_rgb (1) NDWI (1) Nir (1) Red (NIR dataset) (1) Red (RGB dataset) (1)
	Shape	0.25		
	Compactness	0.2		
